# Supplementary material for: The interactome of intact mitochondria by cross-linking mass spectrometry provides evidence for coexisting respiratory supercomplexes
Source: Mol Cell Proteomics. 2017 Dec 8;17(2):216–32. doi: 10.1074/mcp.RA117.000470 (PMC5795388; doi:10.1074/mcp.RA117.000470)
Supplement: Supplemental Data [file supp_RA117.000470_133922_0_supp_23978_fzffwf.zip › spectra_annotation/mito_DR_spectra_annotation/298-1-7-1-8-1.pdf]

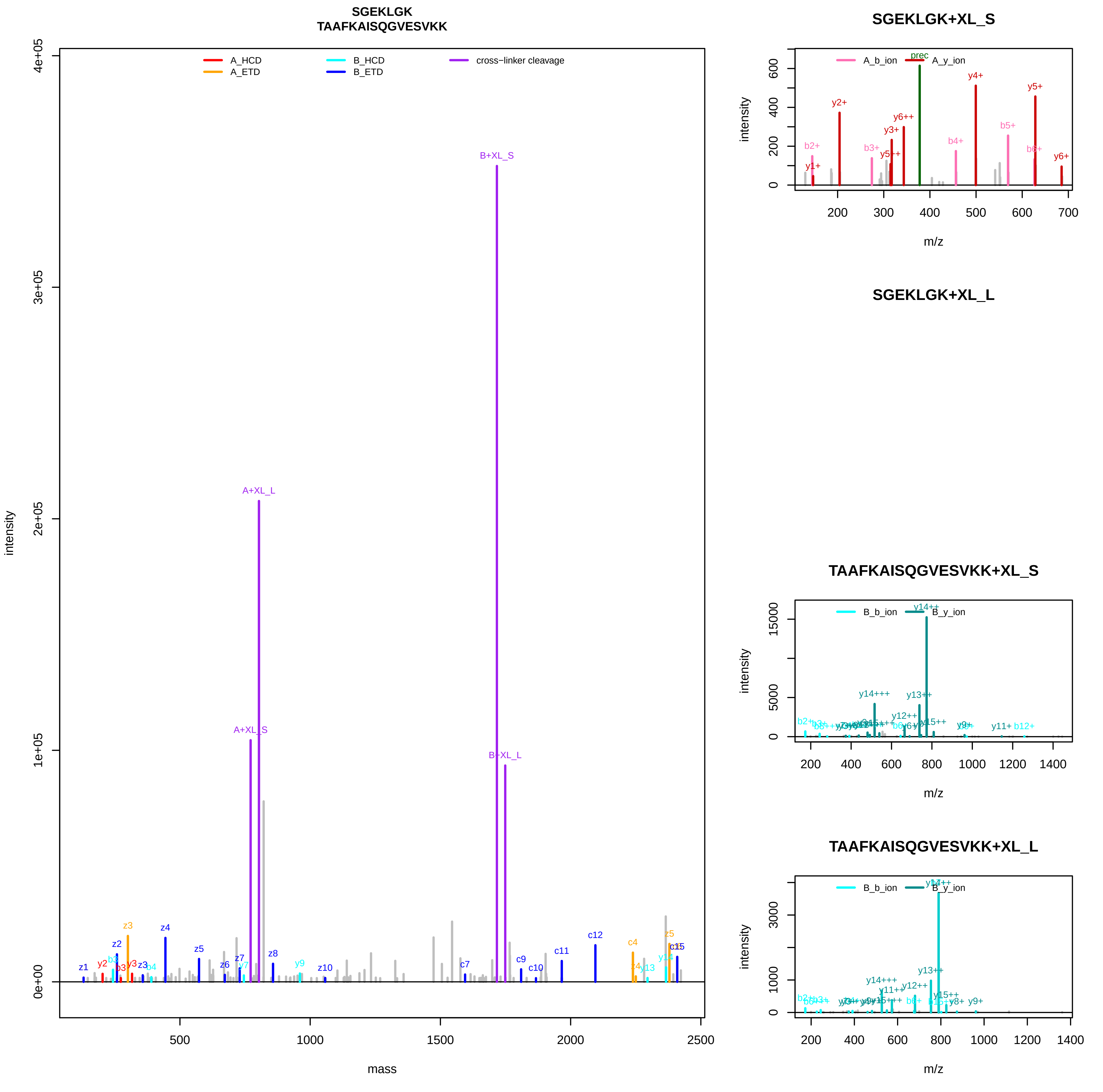
SGEKLGK
TAAFKAISQGVESVKK
intensity
mass
A_HCD
A_ETD
B_HCD
B_ETD
cross−linker cleavage
0e+00
1e+05
2e+05
3e+05
4e+05
500
1000
1500
2000
2500
B+XL_S
A+XL_L
A+XL_S
B+XL_L
z1
y2
b3
z2
y3
z3
b4
z4
z5
z6
z7
y7
z8
y9
z10
c7
c9
c10
c11
c12
c4
y13
y14
z5
c15
SGEKLGK+XL_S
A_b_ion
A_y_ion
prec
intensity
m/z
0
200
400
600
700
y1+
b2+
y2+
b3+
y3+
y5++
y6++
b4+
y4+
b5+
y5+
b6+
y6+
SGEKLGK+XL_L
TAAFKAISQGVESVKK+XL_S
B_b_ion
B_y_ion
intensity
m/z
0
5000
15000
200
400
600
800
1000
1200
1400
y14++
y14+++
y13++
y12++
y15++
y9+
y11+
b12+
b2+
TAAFKAISQGVESVKK+XL_L
B_b_ion
B_y_ion
intensity
m/z
0
1000
3000
200
400
600
800
1000
1200
1400
y14++
y13++
y14+++
y11++
y12++
y15++
y9+
y8+
b6+
b2+
